# Deep-Ultraviolet AlGaN/AlN Core-Shell Multiple Quantum Wells on AlN Nanorods via Lithography-Free Method

**DOI:** 10.1038/s41598-017-19047-6

**Published:** 2018-01-17

**Authors:** Jinwan Kim, Uiho Choi, Jaedo Pyeon, Byeongchan So, Okhyun Nam

**Affiliations:** Convergence Center for Advanced Nano Semiconductors (CANS), Department of Nano-Optical Engineering, Korea Polytechnic University (KPU), Sangidaehakro 237, Siheung-si, 429-793 Gyeonggi-do Korea

## Abstract

We report deep ultraviolet (UVC) emitting core-shell-type AlGaN/AlN multiple quantum wells (MQWs) on the AlN nanorods which are prepared by catalyst/lithography free process. The MQWs are grown on AlN nanorods on a sapphire substrate by polarity-selective epitaxy and etching (PSEE) using high-temperature metal organic chemical vapor deposition. The AlN nanorods prepared through PSEE have a low dislocation density because edge dislocations are bent toward neighboring N-polar AlN domains. The core–shell-type MQWs grown on AlN nanorods have three crystallographic orientations, and the final shape of the grown structure is explained by a ball-and-stick model. The photoluminescence (PL) intensity of MQWs grown on AlN nanorods is approximately 40 times higher than that of MQWs simultaneously grown on a planar structure. This result can be explained by increased internal quantum efficiency, large active volume, and increase in light extraction efficiency based on the examination in this study. Among those effects, the increase of active volume on AlN nanorods is considered to be the main reason for the enhancement of the PL intensity.

## Introduction

Aluminum nitride (AlN) based devices operating in the deep ultraviolet (UVC) regime (200–280 nm) have attracted considerable attention owing to their wide range of potential applications such as medical therapy, UV curing, biochemical sensing, water or air purification, and disinfection^1–3^. However, AlN based UVC devices still suffer from low quantum efficiency and output power. Although the external quantum efficiency of UVC light-emitting diodes (LEDs) has increased dramatically lately, most of the reported efficiency values remain at a few percent of the commercially required level. The low internal quantum efficiency due to a high dislocation density^4,5^ and polarization coefficient^6,7^ is the bottleneck for high-performance UVC applications.

Such critical obstacles can be surmounted by introducing three-dimensional (3D) nanostructures because of their high stress relaxation, low dislocation density, increased active volume, and high extraction efficiency^8,9^. However, AlN based nanostructures for UVC light emitters have seldom been investigated because of the difficulty of using patterned masks. Selective area growth is strongly inhibited because Al atoms have very high sticking coefficient. Zhao *et al*. recently reported that these difficulties can be addressed by employing nitrogen (N)-polar Al(Ga)N nanowires grown on a Si substrate using a lithography-free process^10^. They developed self-organized AlN nanowire LEDs operating at 210 nm, the shortest wavelength ever reported for a nanostructure, and also reported an AlN nanowire laser diode fabricated by radio-frequency plasma-assisted molecular beam epitaxy (MBE)^9,11^. However, AlN or sapphire substrates are preferable for devices operating in the UVC regime because they are transparent to UVC emission. The polarity of the AlN layer can be controlled by varying the preflow conditions before AlN growth and the annealing temperature of the sapphire substrate^12^. Increasing the trimethylaluminum (TMA) preflow rate and decreasing the growth temperature change the crystallographic polarity of the AlN layers from N-polarity to Al-polarity. The step edges of the vicinal sapphire substrate are highly corrugated when the annealing temperature is increased, so nanostructures with different shapes can be fabricated by changing the TMA preflow rate and growth temperature. The surface diffusion coefficient can be written as D = D_0_ exp(−E/*kT*), where E is the energy barrier for hopping, and T is the growth temperature; this equation suggests that the migration length of Al atoms is determined by the growth temperature. For instance, a wall-shaped AlN structure can be grown at higher temperatures than those required to produce a rod-shaped AlN structure because the migration length of Al atoms becomes sufficient to reach the step edges as the temperature is increased. However, the growth process in this method is highly limited because of the very high growth temperature (1350 °C). Here, we present a catalyst/lithography-free sophisticated method for the fabrication of AlN nanorods by polarity-selective epitaxy and etching (PSEE). Then, core-shell-type UVC emitting MQWs grown on AlN nanorods which is formed on sapphire substrate is reported for the first time. In order to develop simple growth process for the fabrication of AlN nanorods, we have only controlled annealing temperature prior to the deposition of LT-buffer layer. In addition, lower growth temperature compared to our previous study provided high density AlN nanorods with better reproducibility.

## Result

### AlN nanorod fabrication by PSEE and AlGaN/AlN MQW growth

**Figure 1:**
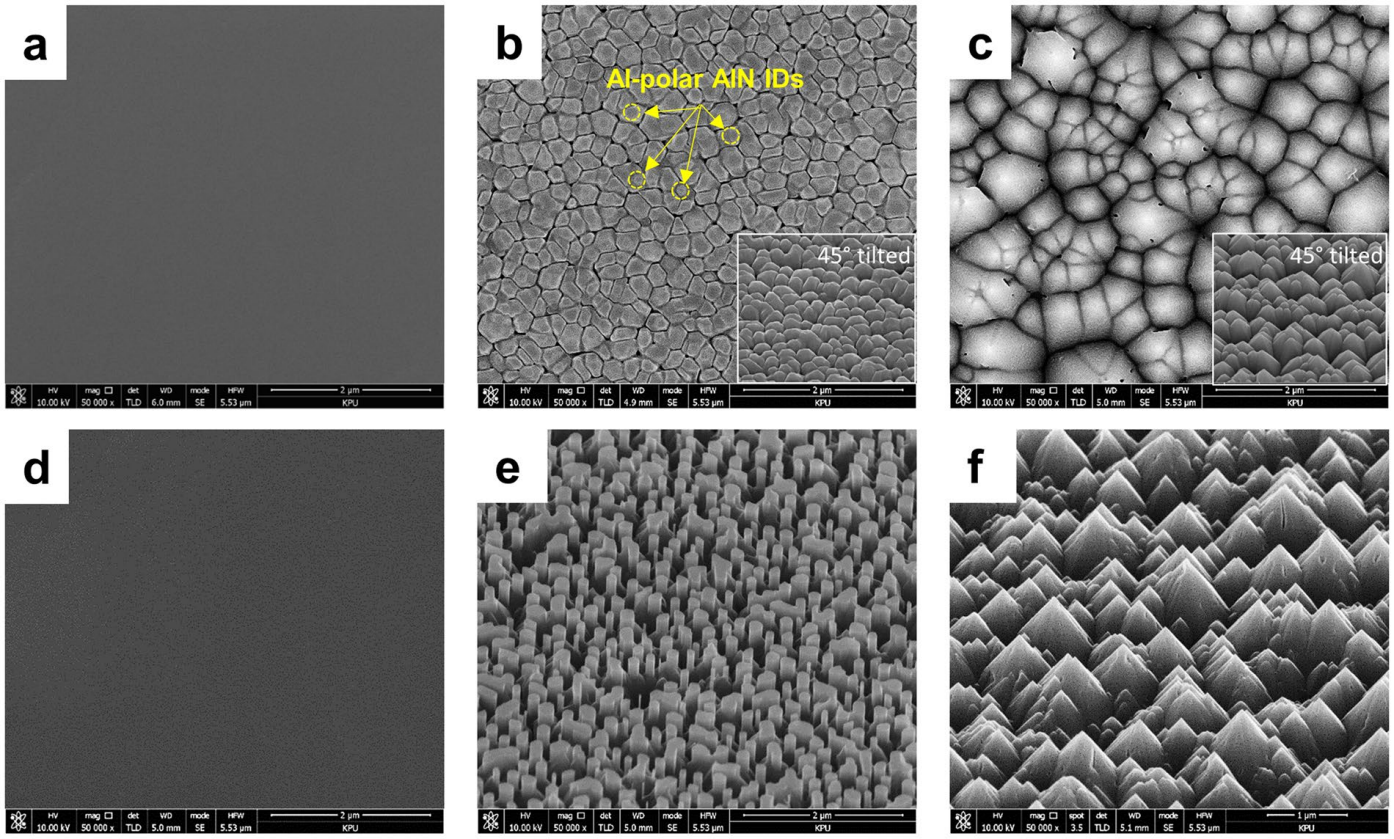
Surface morphology of as-grown AlN templates. Plan-view and 45°-tilted SEM images of surfaces of as-grown and wet-etched AlN layers annealed at (**a**,**d**) 1000 °C, (**b**,**e**) 1100 °C, and (**c**,**f**) 1200 °C, respectively.

Figure 1a–c show plan-view scanning electron microscopy (SEM) images of the as-grown AlN layers. The surface morphology became rough as the annealing temperature increased. This morphological difference is well known to be closely associated with the crystallographic polarity of the AlN layer. The AlN layer obtained at an annealing temperature of 1000 °C (sample A) has a clean surface and is featureless, indicating that it has Al-polarity. The AlN layers obtained at annealing temperatures of 1100 °C (sample B) and 1200 °C (sample C) have columnar morphology with a column width of approximately 300 nm. To clarify the difference in the surface morphology, the as-grown AlN layers were subjected to wet etching in a 0.5 wt% tetramethylammonium hydroxide (TMAH) solution. TMAH solution was chosen as an etchant because it has significantly lower etch rate on nitride than potassium hydroxide (KOH) solution, meaning sophisticated way to control the structure shape. Wet chemical etching was followed by rinsing with deionized water and N_2_ blowing to dry the samples. The morphology of an etched surface provides important information on the polarity because an N-polar surface has a higher etch rate than an Al-polar surface under the same wet chemical etching conditions owing to the difference in bonding conditions between the (0002) and (000-2) planes^13–16^. The lack of significant etching of sample A indicates clearly that the AlN layer has Al polarity, as shown in Fig. 1d. Figure 1f shows that wet etching of sample C resulted in pyramid-shaped hexagonal hillock structures, indicating that the AlN layer has N polarity. Figure 1e shows a noticeable feature of sample B after etching: Columnar Al-polar inversion domains (IDs) appear in the center of the pyramid-shaped hexagonal N-polar AlN domains. Randomly distributed Al-polar IDs were reported by Hussey *et al*.^17^, who grew an AlN lateral polarity structure to form both Al-polar and N-polar AlN layers simultaneously on a sapphire substrate. Their study investigated the origin of Al-polar IDs in N-polar AlN from the interface and attributed them directly to decomposition of sapphire in the presence of hydrogen during film growth. Hydrogen can decompose sapphire and form gaseous Al and water on the surface. Then transformation of the sapphire surface to N-polar AlN is finally possible after a nitridation step. However, the density and size of the Al-polar IDs were not characterized sufficiently because that study focused only on the correlation between sapphire decomposition and ID formation. And in our experiments, nitridation step right after annealing sapphire surface decreased the density of Al-polar IDs. In order to obtain enough density of Al-polar IDs, we simultaneously turned on trimethyl aluminum (TMA) precursors and ammonia. To form light-emitting structures on AlN nanorods in the present study, Al-polar AlN IDs were formed on the sapphire substrate with high density by optimizing the annealing temperature and time.

**Figure 2:**
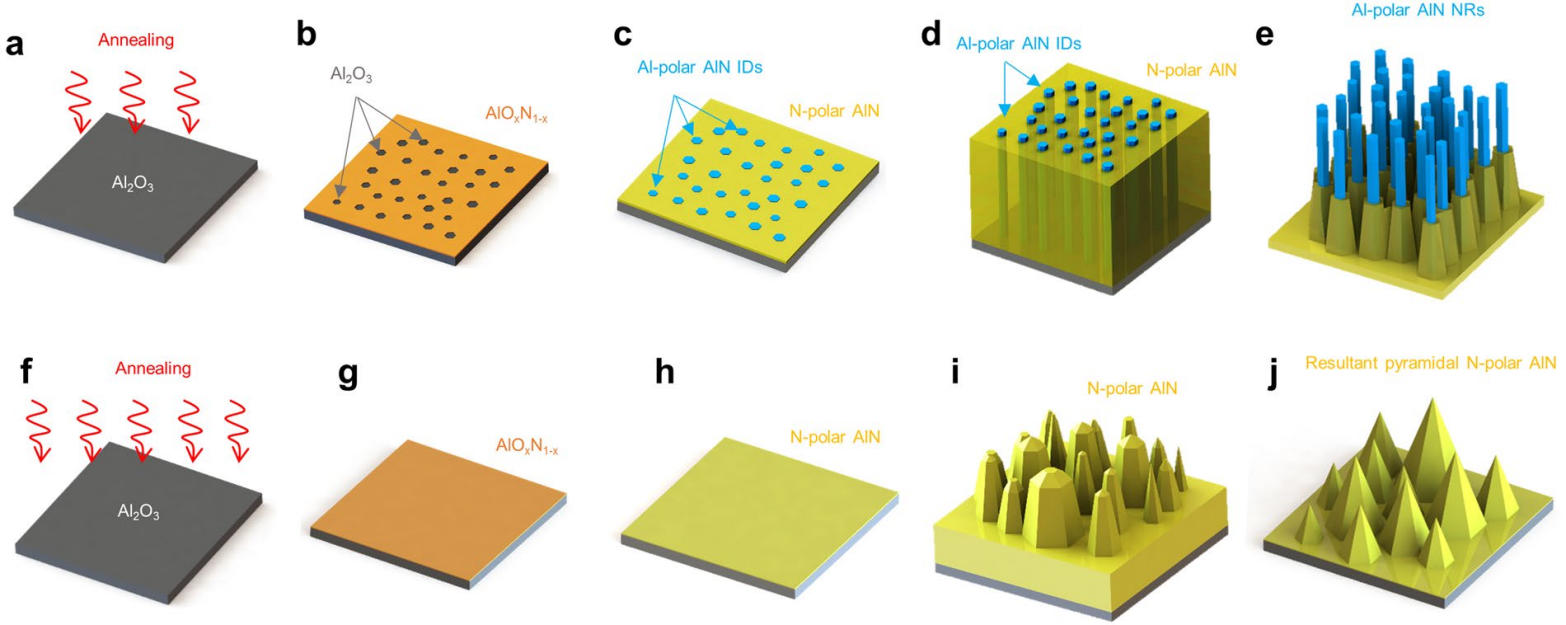
Schematic description of the PSEE process. (**a**) The surface of a sapphire substrate is annealed at 1100 °C. (**b**) An AlO_*x*_N_1−*x*_ layer is formed by oxygen decomposition from sapphire, and regions of bare sapphire exist. (**c**) AlN is deposited on the surface at low temperature, and its polarity is inverted to N polarity on the AlO_*x*_N_1−*x*_ layer. (**d**) A mixed-polarity AlN template is grown at elevated temperature. (**e**) Al-polar AlN nanorods are fabricated after wet chemical etching. (**f**) The surface of the sapphire substrate is annealed at 1200 °C. (**g**) The AlO_*x*_N_1−*x*_ layer entirely covers the surface of the sapphire substrate without openings. (**h**) Only an N-polar AlN layer is grown because there are no openings. (**i**) AlN layer growth at high temperature results in columnar structures with pointed tips. (**j**) Pyramid-shaped N-polar domains remain after wet etching.

Miyagawa *et al*. studied the growth conditions and interface microstructure of AlN on a sapphire substrate by depositing AlN nucleation layers at different temperatures^18^. According to their results, an AlN layer with a nucleation layer grown at 1250 °C had mixed polarity. They found an approximately 50-nm-thick uneven layer on the sapphire substrate, suggesting that a certain chemical reaction occurred during nucleation layer growth and that oxygen atoms from the sapphire diffused into the AlN layer to form an AlO_*x*_N_1−*x*_ phase. Polarity inversion of AlN or GaN by AlO_*x*_N_1−*x*_ on a sapphire substrate was also reported by Wu *et al*. and Wong *et al*.^19,20^. The existence of an AlO_*x*_N_1−*x*_ interlayer between AlN and the sapphire substrate caused the AlN near the interface to have mixed polarity. Here, we describe the PSEE process for the fabrication of Al-polar AlN nanorods. Figure 2a–e show schematic images of PSEE of sample B. Oxygen atoms are diffused from the sapphire substrate. However, the annealing temperature of 1100 °C is not sufficient to form an AlO_*x*_N_1−*x*_ layer on the entire sapphire surface. Therefore, there are randomly distributed openings on which no AlO_*x*_N_1−*x*_ forms because no oxygen in the sapphire substrate decomposes, as shown in Fig. 2a and b. Then, AlN growth is initiated at a lower temperature (950 °C) to deposit a uniform AlN layer on the openings without further decomposition of oxygen from the sapphire substrate. After the seed AlN layer is deposited, the growth of an AlN layer at high temperature (1200 °C) provides high crystal quality and a rough surface owing to the faster growth rate of Al-polar IDs^21^, as shown in Fig. 2d. Etching of sample B produces Al-polar IDs located in the center of the pyramid-shaped N-polar AlN domains. Wet etching of nitride is well known to be polarity-selective, as the N-polar surface reacts and is easily etched, whereas the Al-polar surface shows no reaction. Further, the *m*-faceted surfaces of the resultant Al-polar AlN IDs are more reactive than the *c*-plane surfaces, so Al-polar AlN nanorods surrounded by N-polar AlN domains are formed under appropriate etching conditions. On the other hand, only an N-polar AlN layer is grown after high-temperature annealing because oxygen diffuses out from the entire sapphire surface. Oxygen decomposition forms an AlO_*x*_N_1−*x*_ layer without any openings, as shown in Fig. 2g and h. The high-temperature growth of AlN on the entire AlO_*x*_N_1−*x*_-covered surface produces columnar N-polar AlN with a sharply pointed apex. The columnar N-polar AlN layer leaves pyramid-shaped N-polar AlN domains after wet chemical etching, as shown in Fig. 2i and j. Thus, in order to form nanorods on sapphire substrate, we only need to know proper annealing temperature before nucleation layer deposition to successfully fabricate Al-polar AlN IDs, without using lithography.

**Figure 3:**
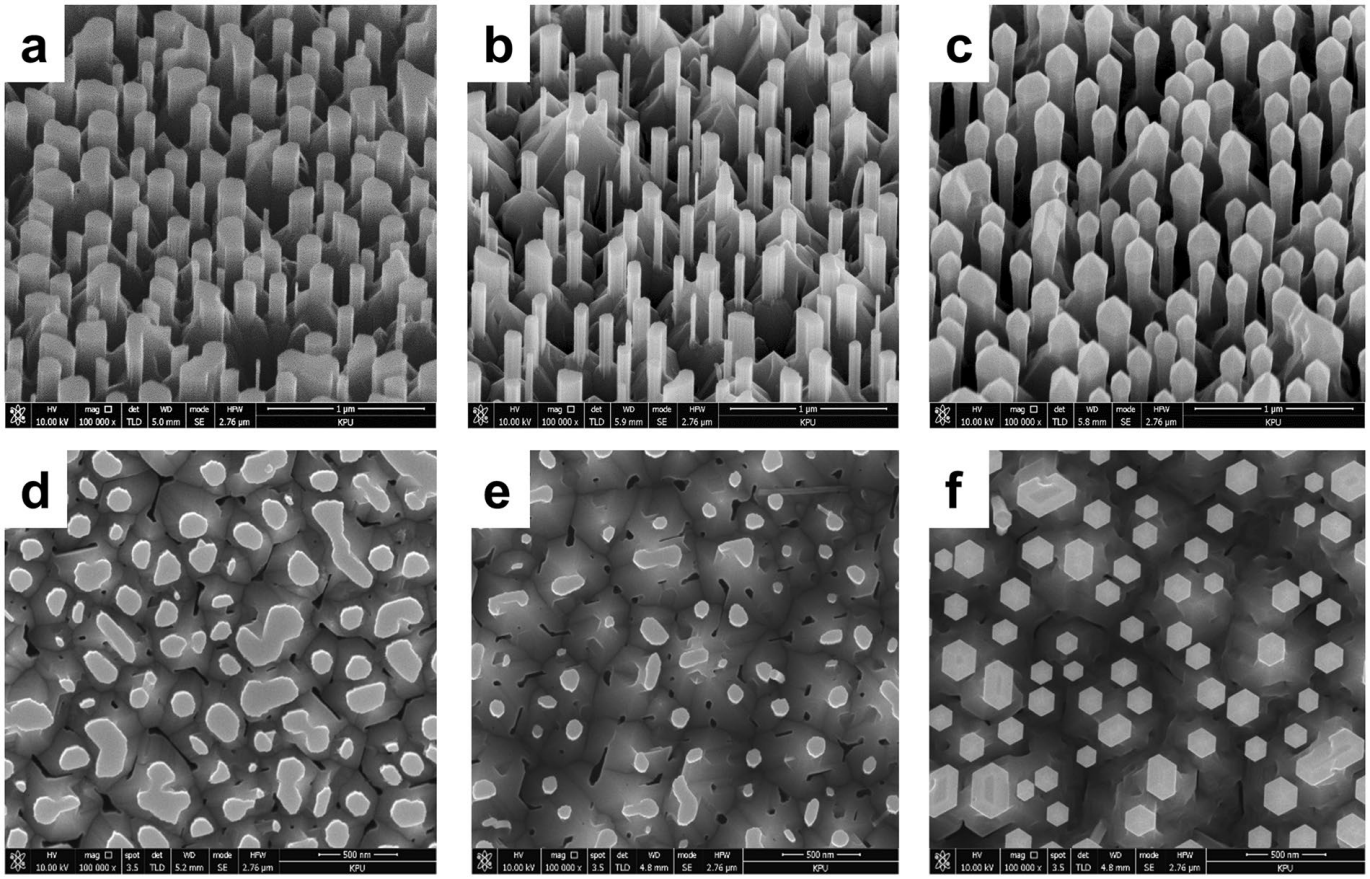
Typical bird’s-eye and plan-view SEM images of mixed-polarity AlN template after wet chemical etching. (**a**,**d**) 20 min and (**b**,**e**) 40 min. (**c**,**f**) Images of regrown MQWs on the AlN nanorods etched for 40 min.

The diameter of the Al-polar AlN nanorods can be controlled by wet chemical etching in the PSEE process. Figure 3a,d and b,e show SEM images of sample B after wet chemical etching for 20 and 40 min, respectively. The wet etching condition was adjusted to be slower than that in our previous study to precisely control the etching rate of the AlN nanorods. The average diameter of the AlN nanorods, obtained from plan-view SEM images, decreased from 180 to 95 nm as the etching time increased, and the density of the AlN nanorods decreased slightly from 6.8 × 10^−8^ cm^−2^ to 6.2 × 10^−8^ cm^−2^ because AlN nanorods with smaller diameters were etched out and disappeared. After 20-nm-thick AlN and five periods of Al_0.57_Ga_0.43_N/AlN multiple quantum wells (MQWs) were regrown on the AlN nanorods, interesting behavior is observed, as shown in Fig. 3c. The regrown Al_0.57_Ga_0.43_N/AlN MQWs expand both laterally and vertically from the fabricated AlN nanorod template and exhibit not only (1–100) *m*-plane sidewalls but also inclined semipolar facets on top of the nanorods. The formation of sixfold (1–100) *m*-plane facets reduces the irregularity of the final shape, as shown in Fig. 3f, owing to a facet recovery growth process previously reported for core–shell GaN nanostructures^22^. The side facets on top of the AlN nanorods had an angle of approximately 62° from the horizontal, indicating that they are on the (10–11) semipolar plane. As growth proceeds, the (0002) *c*-plane facet region shrinks, and pyramids can exist only on the top surface. The progressive reduction of the top *c*-plane facet by the formation of six (10–11) semipolar side facets is closely related to the different growth rate of each facet because still-growing facets with the lowest growth rate are visible after growth^23,24^. At the early stage of growth, vertical growth of nanorods is restrained due to a strong consumption of Al atoms in forming semipolar sides. Later, Al atoms which can not reach the bottom side m-plane border start to contribute toward vertical growth. The compact nanorods will have smaller perimeter so that start vertical growth faster than bigger nanorods. Besides, larger initial area of c-plane allows bigger nanorods to last longer although they have higher c-plane reduction rate than smaller nanorods. As a result, earlier disappearance of c-plane area in smaller nanorods is observed. The (10–11) semipolar plane has a slower growth rate than the *c*-plane because of H_2_ passivation. It is well known that the carrier gas plays a crucial role in determining the quality of the grown surface, and H_2_ is commonly used in Al(Ga)N growth by metal–organic chemical vapor deposition (MOCVD). Several studies have proved that H_2_ can passivate a surface that is terminated by nitrogen^25–27^. According to a ball-and-stick model, the atomic structure of the semipolar (10–11) plane surface is terminated with nitrogen atoms when the top surface of the grown structure is terminated with III-polar atoms^28^. Thus, we can assume that the strong N–H bonds are passivated, stabilizing the semipolar surface in H_2_ ambient. The atomic structure and growth mode of the regrown AlN and MQWs are discussed in the next section. The (10–11) plane surface under H_2_ passivation can rarely accommodate Al and Ga atoms, and this leads to a lower growth rate. This phenomenon can also explain why the upper parts of some AlN nanorods with large diameters have a truncated pyramidal shape, whereas AlN nanorods with small diameters exhibit a perfect pyramidal shape. The narrower AlN nanorods have a compact *c*-plane area; therefore, the top (0002) surface is reduced more rapidly than that of the wider nanorods. Accordingly, it is suggested that the shape of the regrown AlN nanostructure is determined by competition between various crystal planes that have different growth rates.

### Optical characterization of AlGaN/AlN MQWs

**Figure 4:**
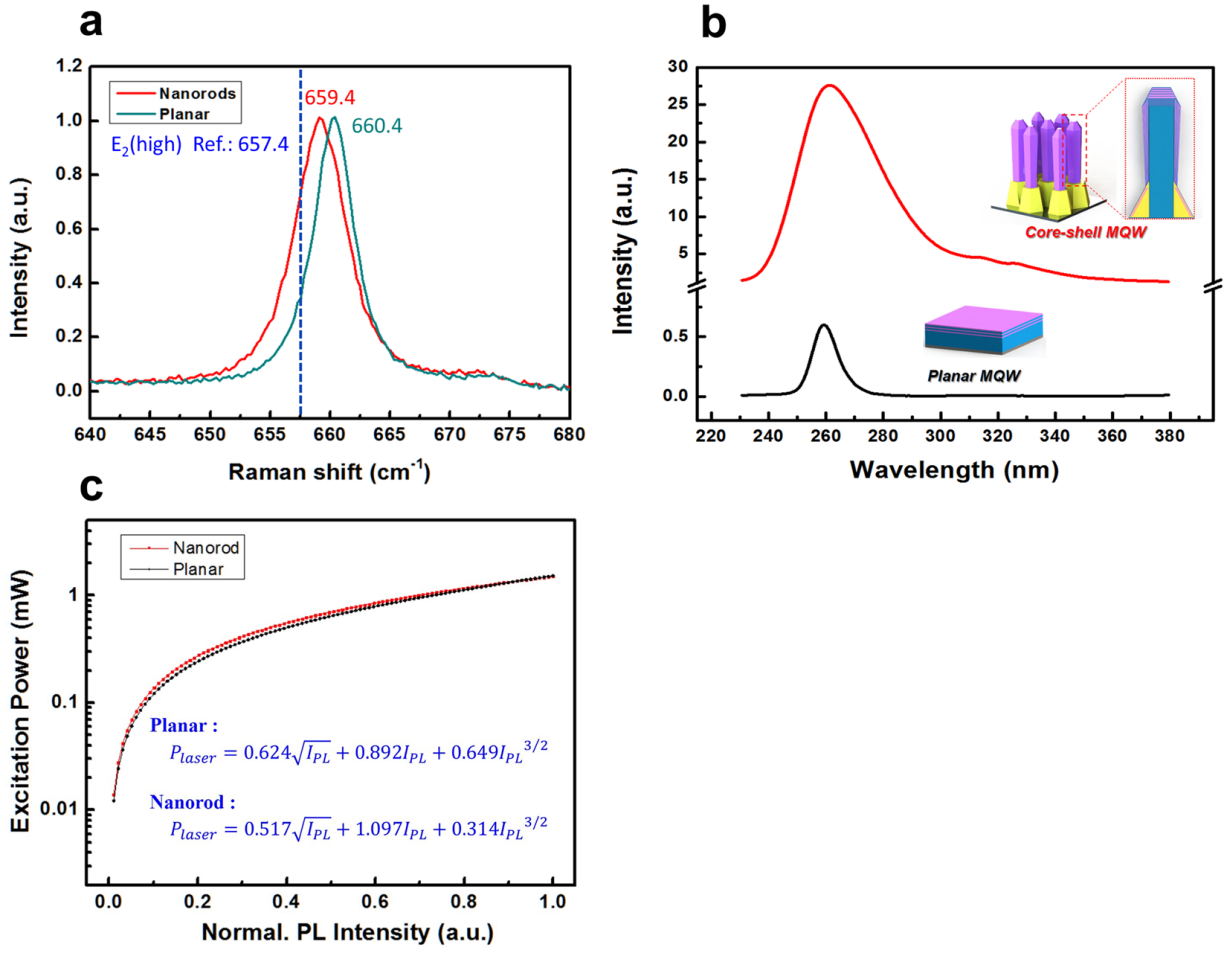
Optical characterization of fabricated AlN nanorods and regrown AlGaN/AlN MQWs. (**a**) Raman spectra of fabricated AlN nanorods. (**b**) PL spectra of AlGaN/AlN MQWs subsequently grown on the AlN nanorods and on a conventional planar layer. (**c**) Fitted curves of integrated PL intensity as a function of laser excitation power obtained from Eq. (4).

Figure 4a shows the Raman spectra of the AlN nanorods and the planar AlN layer measured using the 632.8 nm line of a He-Ne laser as the excitation source. The phonon frequency of the unstrained AlN layer at room temperature is 657.4 cm^−1^ for the E_2_ (high) phonon mode. The strongest E_2_ (high) phonon frequencies of the AlN nanorods and planar AlN layer are 659.4 and 660.4 cm^−1^, respectively. The shift of the Raman spectra to the higher-frequency side compared with that of the unstrained AlN indicates the existence of biaxial compressive strain due to mismatches in the lattice constant and thermal expansion coefficient between AlN and the sapphire substrate. However, the E_2_ (high) phonon mode of the AlN nanorods was shifted slightly toward the lower-frequency side and is close to the unstrained frequency of AlN. This slight shift is attributed to size effects of the 3D nanostructure, and AlN nanorods could be a strain-relaxed template for the upper structure layers. The slight broadening in Raman peak of AlN nanorods sample can be explained by disparity in its atomic composition, crystal size, molecular chain length, and morphology compared to planar structure.

Figure 4b shows the photoluminescence (PL) spectra of MQWs grown on the nanorods and on the planar structure, which were obtained using a 213 nm pulsed laser. The peak wavelength of the planar MQWs and core–shell MQWs are 259 and 261 nm, respectively. The broadening in the PL peak of the core–shell MQWs is due to compositional fluctuation of adatoms with different diffusion lengths on the different facets and irregular gas delivery to the nanostructure^29^. The PL intensity of the MQWs grown on the AlN nanorods was approximately 40 times higher than that of simultaneously grown planar MQWs. There are several reasons for this dramatic enhancement of the emission intensity. First, the defect density is well known to decrease greatly when a nanostructure is employed. In this case, the mixed-polarity AlN template was grown by a bottom-up approach, and the AlN nanorods were fabricated by top-down wet chemical etching. Wet chemical etching preferentially etches defects, unlike mechanical etching. Therefore, MQWs could be grown on the defect-reduced AlN nanorods in this study. Furthermore, the sixfold (1–100) *m*-plane facets of the sidewalls can help suppress the polarization field effect, which significantly decreases the internal quantum efficiency^30^. A comprehensive analysis of the polarization effect in an Al_0.5_Ga_0.5_N template has been reported. The calculation results indicated that the piezoelectric polarization field becomes nearly zero for nonpolar and semipolar orientations^31^. Therefore, MQWs grown on the *m*-plane facet naturally have a high internal quantum efficiency.

There are diverse ways to determine internal quantum efficiency (IQE) of MQWs such as temperature dependent PL measurements, time-resolved PL measurements, and excitation power dependent PL measurements. Bryan *et al*., reported that the results from power dependent PL measurement using SRH model was shown for a fair comparison with previous reports^32,33^. The IQE in this study was calculated using the model based on rate equation which is proposed by Yoo *et al*.^34^. The proposed model is based on the rate equation, which is the relationship between total carrier generation *(G)* and individual rate

1 $${\rm{G}}={\rm{An}}+{{\rm{Bn}}}^{2}+{{\rm{Cn}}}^{3}$$

IQE is defined as the radiative recombination rate over the total carrier generation rate,

2 $${\rm{IQE}}=\frac{{{\rm{Bn}}}^{2}}{{\rm{An}}+{{\rm{Bn}}}^{2}+{{\rm{Cn}}}^{3}}$$

The terms in the formula of proposed model uses fitting parameter *P*_1_, *P*_2_, and *P*_3_ as follows:

3 $${{\rm{P}}}_{{\rm{laser}}}={{\rm{P}}}_{1}\sqrt{{{\rm{I}}}_{{\rm{PL}}}}+{{\rm{P}}}_{2}{{\rm{I}}}_{{\rm{PL}}}+{{\rm{P}}}_{3}{{{\rm{I}}}_{{\rm{PL}}}}^{3/2}$$

And IQE can be directly calculated from the following equation:

4 $${\rm{IQE}}=\frac{{{\rm{Bn}}}^{{\rm{2}}}}{{\rm{G}}}=\frac{{{\rm{I}}}_{{\rm{PL}}}{{\rm{P}}}_{{\rm{2}}}}{{{\rm{P}}}_{{\rm{laser}}}}$$

In order to change the carrier density in the active region, the laser power was tuned from 0.015 mW to 1.5 mW using neutral density filters and we successfully obtained fitting parameters *P*_2_ for MQWs on both planar and nanorod structures from the fitting curves as shown in Fig. 4c.

MQWs on planar:

5 $${{\rm{P}}}_{{\rm{laser}}}=0.624\sqrt{{{\rm{I}}}_{{\rm{PL}}}}+0.892{{\rm{I}}}_{{\rm{PL}}}+0.649{{{\rm{I}}}_{{\rm{PL}}}}^{3/2}$$

MQWs on nanorod:

6 $${{\rm{P}}}_{{\rm{laser}}}=0.517\sqrt{{{\rm{I}}}_{{\rm{PL}}}}+1.097{{\rm{I}}}_{{\rm{PL}}}+0.314{{{\rm{I}}}_{{\rm{PL}}}}^{3/2}$$

The IQEs calculated using Eq. (4) are 73% and 59% for the MQWs on nanorods and the MQWs on planar AlN at the highest excitation (1.5 mW) and the increased IQE of the MQWs on nanorods might be attributed to the reduced defect density and relieved strain.

Next, we would like to point out that core–shell-type MQWs have a large active volume with a minimized polarization field due to the (10–10) *m*-plane facets. Core–shell-type MQWs are typically grown by MOCVD, whereas MBE is suitable only for fabricating uniaxial nanostructure, and controlled growth of a coaxial nanostructure by MBE has not been reliably demonstrated. The primary advantage of a core–shell-type active region over uniaxial and conventional planar-type MQWs is the potentially large active volume grown on the side facets of nanorods, which can greatly increase the output power. It is reasonable to assume that this increased active volume is the main reason for the enhancement in PL intensity. A substantial improvement in PL intensity from the nanorods compared to planar sample was observed in InGaN/GaN MQWs on nanorods and the study also reported that when the nanorod’s diameter increases, the luminescence intensity is also increased^35^.

Finally, it is also important to note that the 3D nanostructure results in increased light extraction efficiency. According to a calculation in a simulation using LightTools (shown in the Supplementary Information), the light extraction efficiency (LEE) increased from 0.31 to 0.45 after the nanostructure was employed. This increase in the LEE is due mainly to a reduction in the total internal reflection. Those values may have been exaggerated because it was a simple numerical calculation using ray-trace method and TM-polarized profile of emitting light was not considered in the calculation. Light re-absorption in the 3D structure also can decrease extraction efficiency. However, an attempt was made to briefly discuss the extraction efficiency of 3D structure compared to planar structure without any sort of extra roughening process.

### TEM measurements of AlN nanorods with AlGaN/AlN MQWs

**Figure 5:**
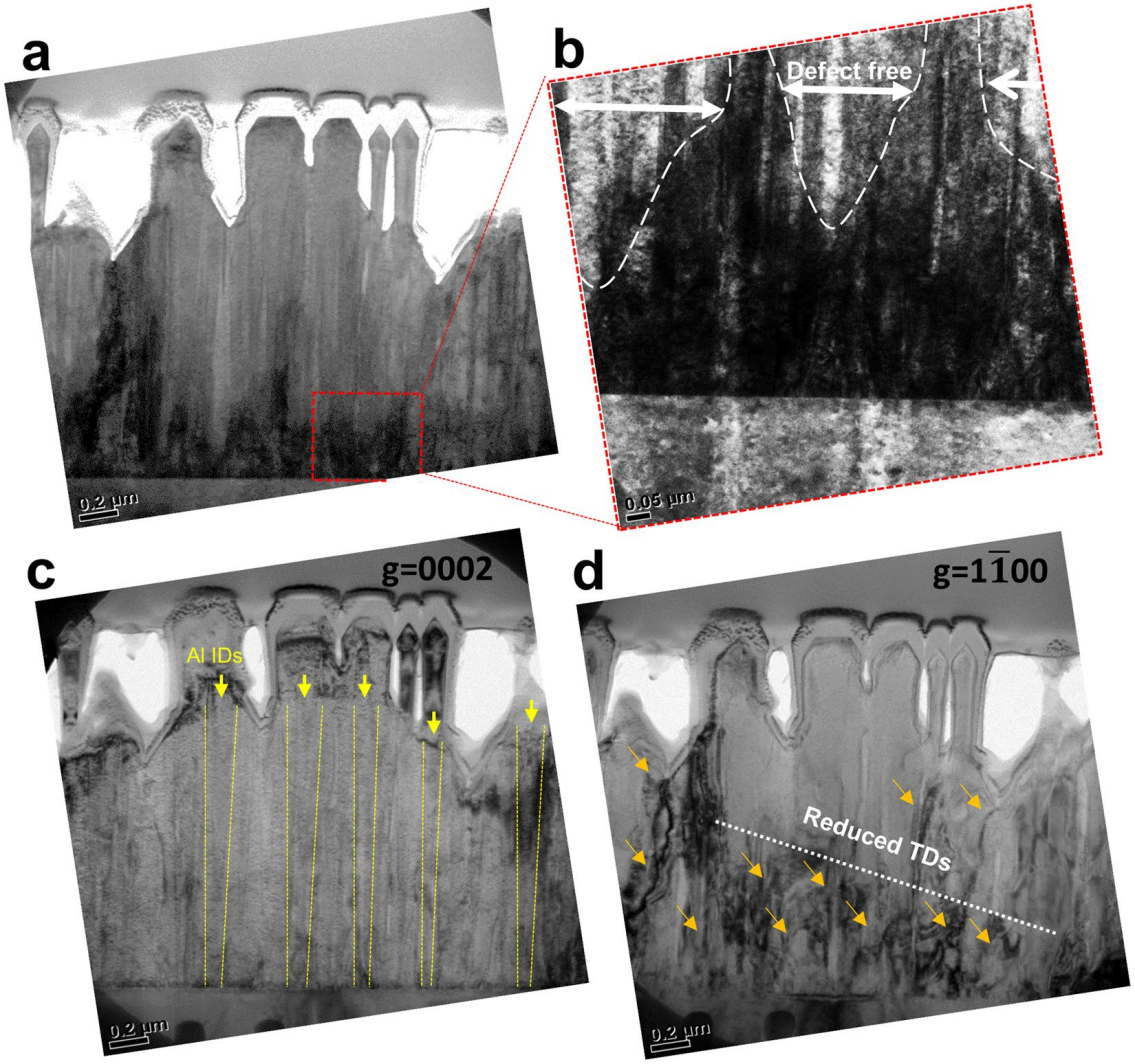
Cross-sectional TEM images of regrown AlGaN/AlN MQWS on mixed-polarity AlN nanorods. (**a**) STEM images of nanorods. (**b**) Magnified image of area in red dashed box in (**a**); annihilation of dislocations within domain boundaries is observed. (**c**) ID boundaries are observed along (0002) g vector. (**d**) TEM image along (1–100) g vector; edge-type dislocations are gathered and finally terminated inside neighboring N-polar AlN domains.

To evaluate the defect reduction behavior in the mixed-polarity template, bright-field transmission electron microscopy (TEM) analysis was conducted. Figure 5a shows cross-sectional TEM images of AlN nanorods through the [11–20] zone axis, and Fig. 5b shows a magnified image of the region enclosed by the red dashed square in Fig. 5a. The threading dislocations (TDs) generated from the surface of the sapphire substrate are gathered within boundaries between separated N-polar AlN domains, as shown by the triangular dark region near the bottom. Figure 5b shows that nearly defect-free regions appear more than ~300 nm from the interface between the AlN and sapphire substrate; the reason is bending of dislocations in the adjacent N-polar AlN boundaries. Figure 5c shows that ID boundaries are observed along the (0002) g vector; this finding is consistent with previous studies of AlN and GaN, and Al-polar IDs in the center of N-polar AlN domains are also clearly visible in our grown AlN nanorods. Cross-sectional TEM along the (1–100) g vector was performed to clarify the bending of edge-type TDs, which are the major dislocation in nitride films with a Burgers vector b of (1/3) <11–20>, as shown in Fig. 5d. The edge-type TDs are confined at the interface between the AlN and the sapphire substrate. However, they start to vanish as growth proceeds, and an almost defect-free region appear after the thickness of the AlN layer increases beyond 400 nm. This is attributed to the growth mechanism, in which a mixed-polarity AlN layer with a faceted island shape grows in a 3D growth mode resulting from the different growth rates of Al-polar and N-polar AlN domains. Because TDs tend to bend toward the surface of the faceted islands in 3D growth mode, the regions between N-polar AlN domains surrounding Al-polar IDs become defect-free areas as the thickness of the mixed-polarity AlN layer increases. This phenomenon has been further explained elsewhere^36,37^.

**Figure 6:**
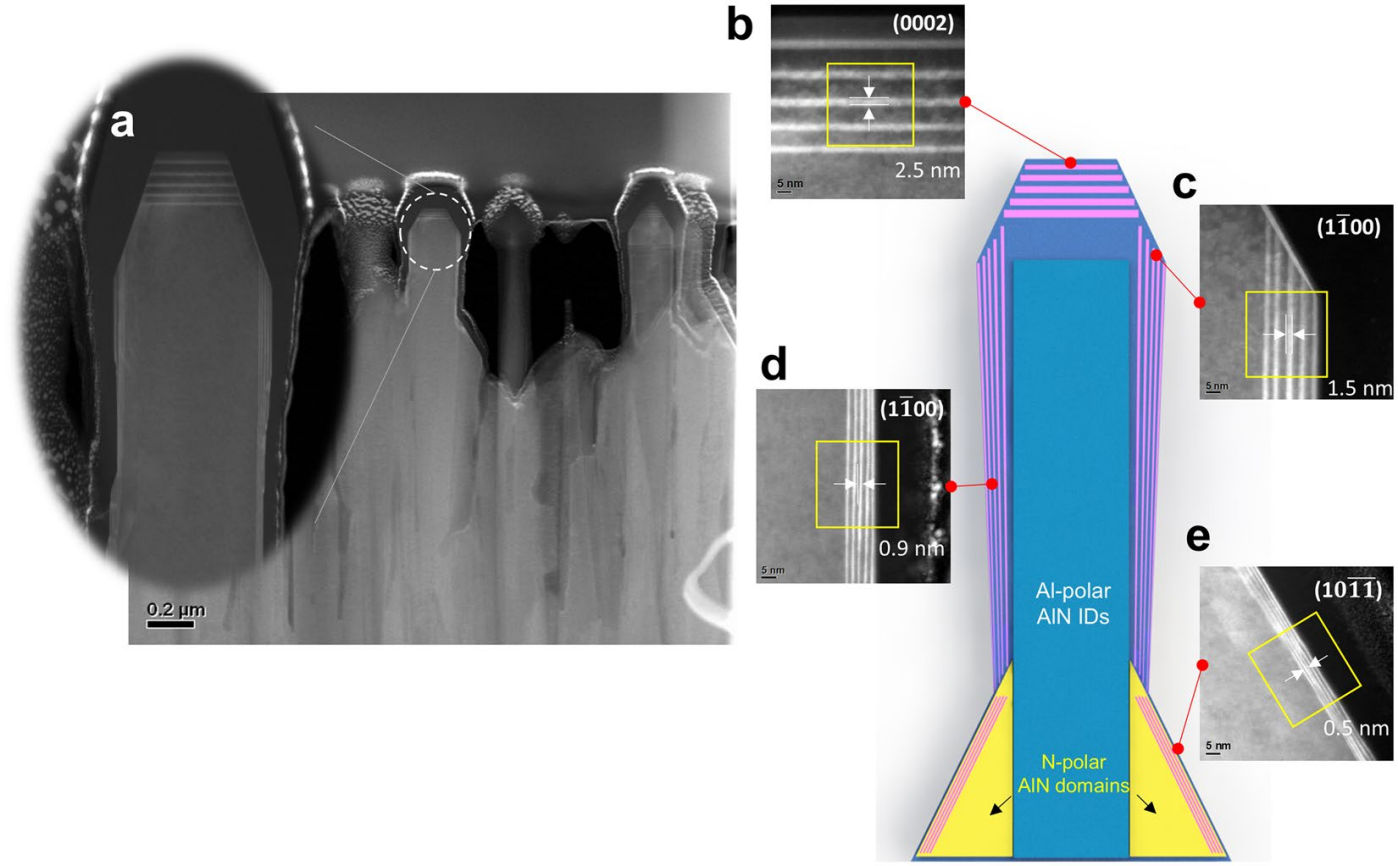
TEM images of regrown AlGaN/AlN MQWs. (**a**) The regrown MQWs consist of three facets. (**b**) On the (0002) *c*-plane facet, which has the highest growth rate, a truncated pyramid shape is grown. (**c**) The (1–100) *m*-plane MQWs grown on the upper part of the nanorods are thicker than those on the lower part. (**d**) The thickness of *m*-plane MQWs is decreased in the lower region. (**e**) The MQWs grown on the (10-1-1) facet are thinner than those on the other facets owing to insufficient reactant delivery and a lower growth rate.

**Figure 7:**
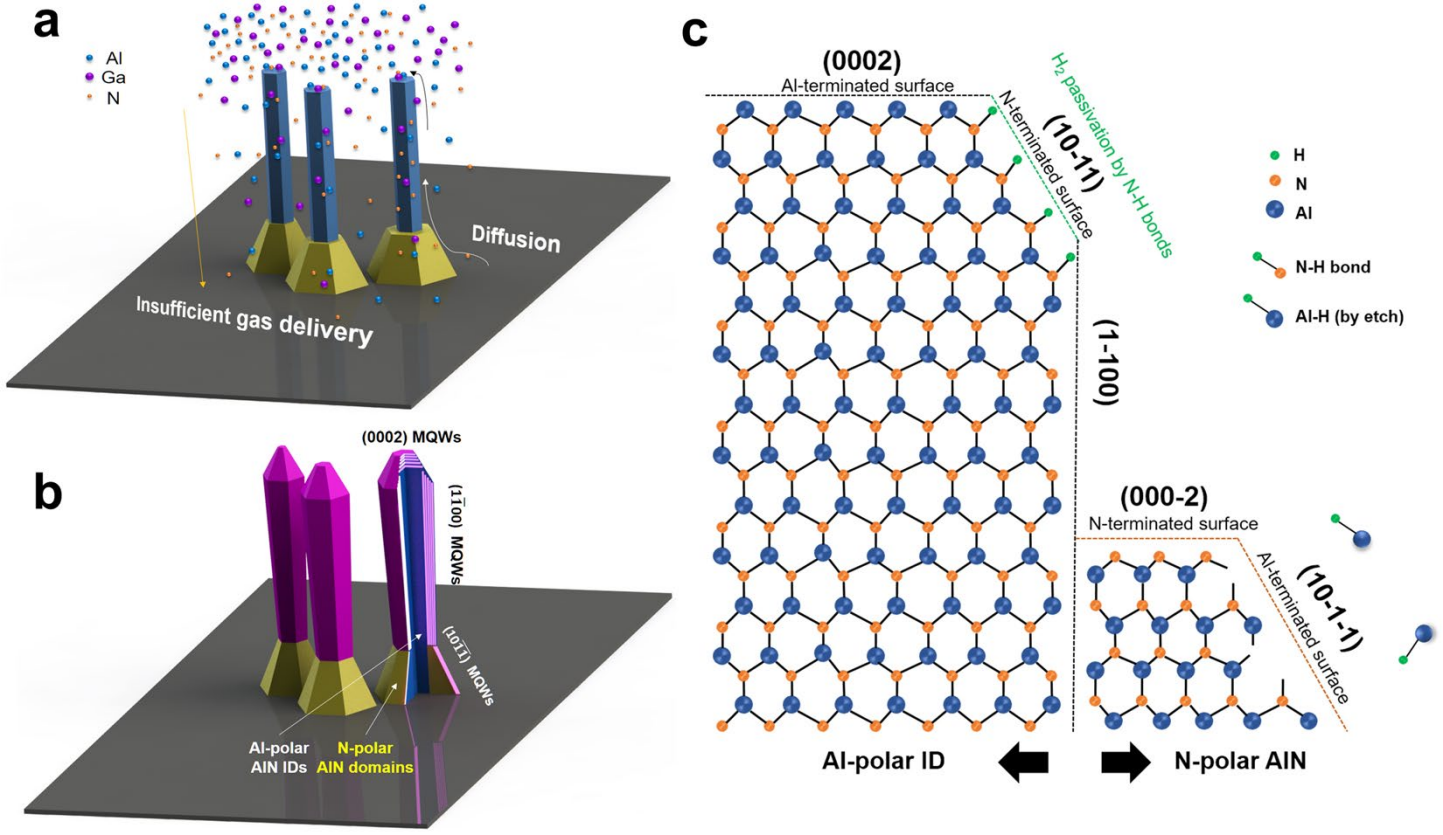
Illustration of growth process of AlGaN/AlN MQWs and atomic structure of Al-polar ID and N-polar AlN domain. (**a**) Precursors come from the top of the AlN nanorods in the reactor. (**b**) The tapered structure of the regrown MQWs is attributed to insufficient gas delivery in the bottom region and different diffusion lengths of Al and Ga atoms on the various planes. (**c**) The (10–11) semipolar plane has the slowest growth rate owing to H_2_ passivation, and regrowth of MQWs is impeded on the surface. The (10-1-1) plane on the N-polar AlN domain is terminated with Al atoms, so growth and etching occur simultaneously.

To obtain detailed information on the regrown MQWs, scanning transmission electron microscopy (STEM) images of cross-sectioned AlN nanorods including five periods of Al_0.57_Ga_0.43_N/AlN MQWs were observed along Z = [11–20]. The STEM images shown in Fig. 6 confirm that MQWs with (0002), (1–100), and (10-1-1) facets are formed on the top, sidewall, and remaining N-polar AlN domain in the bottom region, respectively. The highest growth rate on the (0002) c-plane among the formed planes results in thicker MQWs having 2.5-nm-thick AlGaN wells. The MQWs grown on the (1–100) m-plane sidewall were thinner than those on the c-plane owing to their lower growth rate. This lower growth rate on the (1–100) m-plane is due to III–III (the surface is covered by Group III metallic atoms) or III–N dimers^38,39^. The regrowth process of AlGaN/AlN MQWs is depicted in Fig. 7a and b to explain the irregularity of the MQWs regrown on the side facets. The tapered structure of the regrown MQWs is mainly due to insufficient gas delivery to the bottom region of the nanorods and the different diffusion lengths of Al and Ga atoms on the various facets during growth. The atomic structure of the regrown MQWs on the AlN nanorods is also shown in Fig. 7c to explain the growth mechanism on each facet. According to Lee *et al*. and Waltereit *et al*., III–III (Al–Al and Ga–Ga) dimers are preferentially formed on the (1–100) surface under a III-rich condition, whereas stoichiometric III–N dimers are formed on the (1–100) surface under stoichiometric and nitrogen-rich growth conditions^40,41^. This behavior was studied using a first-principles density functional theory calculation and has been experimentally proved. Both types of dimers passivate the (1–100) facet, reducing the growth rate of the (1–100) m-plane sidewall, and the regrown MQWs in this study are in good agreement with the growth mechanism described above. It is also noteworthy that no MQWs were grown on the (10–11) plane, whereas MQWs were grown on the (10-1-1) plane of the N-polar AlN domain. To explain this result, a ball-and-stick model should again be considered with two coupled processes. The surface of the (10-1-1) plane is terminated with Group III metallic atoms, like the (0002) c-plane surface. Thus, H_2_ passivation of the surface does not occur, and dangling bonds exist on the (10-1-1) surface. Therefore, the H_2_ enhances AlN decomposition, which is strongly affected by the atomic structure of the crystal planes and the growth temperature because H_2_ carrier gas could easily etch the Al or Ga atoms on the (10-1-1) surface via Al–H, Ga–H, and N–H bond formation. At the relatively low temperature used for MQW growth, namely, 1070 °C, the number of adsorbed reactant atoms exceeds that of etched atoms. Under these circumstances, growth has an advantage in its competition with etching, which explains why MQWs were grown on the (10-1-1) surface. This result confirms the previous study of Tian *et al*.^31^.

## Discussion

We realized a lithography-free approach to fabrication of AlN nanorods that combines bottom-up growth of a mixed-polarity AlN template using MOCVD and top-down wet chemical etching by PSEE. It was confirmed that a mixed-polar AlN template could be easily grown by controlling only the annealing temperature before growth process. In addition, we report for the first time regrowth of core–shell-type AlGaN/AlN MQWs on AlN nanorods having three crystallographic orientations with polarization-free facets, which exhibit a dramatically increased PL intensity in the UVC range. As a continuation of the research, we are trying to realize an electrically driven AlGaN nanorods LED by replacing present AlN nanorods to n-AlGaN nanorods. The largest bottleneck is leakage current at the domain boundary between Al-polar ID and residual N-polar AlN. High resistance of n-AlGaN due to wide band gap is also an insuperable difficulty. We are now examining proper shape (perimeter/height) of n-AlGaN for improved current spreading with reduced leakage current and these n-AlGaN nanorods will provide a solution to previous problems related to fabrication of nanostructured UVC LEDs.

## Methods

### Epitaxy of AlN nanorods templates

All the AlN layers were grown on *c*-plane sapphire substrates with an offcut angle of 0.2° along the *m* axis by a RF-heated high-temperature MOCVD system with close-coupled showerhead type reactor (Top Engineering, PHAETHON 100U). Trimethylaluminum (TMA), trimethylgallium (TMG), and ammonia (NH_3_) were used as the Al, Ga, and N precursors, respectively. Before growth, the *c*-plane sapphire substrates were thermally cleaned in H_2_ ambient at 1000, 1100, and 1200 °C with an annealing time of 300 seconds for all samples, respectively. Then a 25-nm-thick AlN nucleation layer and a 2-μm-thick high-temperature AlN layer were deposited. The growth temperatures of the nucleation layer and high-temperature AlN layer were 950 and 1200 °C, respectively. The growth rate and V/III ratio of nucleation layer and HT-AlN template layer were 0.48 μm/h and 2 µm/h at V/III ratio of 2000 and 300, respectively. Then AlGaN/AlN MQWs were grown at 1070 °C with a higher V/III ratio (4000) to minimize the incorporation of point defects.

### Wet chemical etching (Fabrication of AlN nanorods)

The polarity of the grown AlN layers was confirmed by wet chemical etching using an aqueous 0.5 wt% TMAH solution at room temperature. Etching times of 20 and 40 min were selected to control the diameter of the AlN nanorods.

### High-resolution scanning electron microscopy (HR-SEM)

The surface morphology of the AlN layers was observed using 45°-tilted and plan-view images obtained by HR-SEM (FEI Co., Nova Nano SEM 200).

### Raman spectroscopy

Raman spectra were recorded by a Horiba Jobin-Yvon LabRam Aramis spectrometer. The 632.8 nm line of a He-Ne laser was used as the excitation source.

### Photoluminescence (PL)

The PL intensity of MQWs simultaneously grown on a planar structure and on nanorods was measured using a 213 nm pulsed laser (CrysLaS Laser Systems, Q-Series).

### Scanning transmission electron microscopy (STEM)

Cross-sectional TEM images of nanorods were obtained using analytical SEM (JEOL, JEM2100F). The specimen for the TEM measurement was prepared using a focused ion beam milling machine (FEI, NOVA 600 Nanolab).

### Computation of light extraction efficiency

The light extraction efficiency was calculated using the LightTools simulation program.

## Electronic supplementary material

Supplementary Information
