# Supplementary material for: Deep-Ultraviolet AlGaN/AlN Core-Shell Multiple Quantum Wells on AlN Nanorods via Lithography-Free Method
Source: Sci Rep. 2018 Jan 17;8:935. doi: 10.1038/s41598-017-19047-6 (PMC5772499; doi:10.1038/s41598-017-19047-6)
Supplement: Supplementary file 1 — Supplementary Information [file 41598_2017_19047_MOESM1_ESM.doc]

**Supplementary Information

Deep-Ultraviolet AlGaN/AlN Core-Shell Multiple Quantum Wells on AlN Nanorods
via Lithography-Free Method**

Jinwan Kim, Uiho Choi, Byeongchan So, Jaedo Pyeon, and Okhyun Nam*

Convergence Center for Advanced Nano Semiconductors (CANS), Department of Nano-Optical Engineering, Korea Polytechnic University (KPU), Sangidaehakro 237, Siheung-si 429-793, Gyeonggi-do, Korea

*Corresponding author : Okhyun Nam ([ohnam@kpu.ac.kr](mailto:ohnam@kpu.ac.kr))


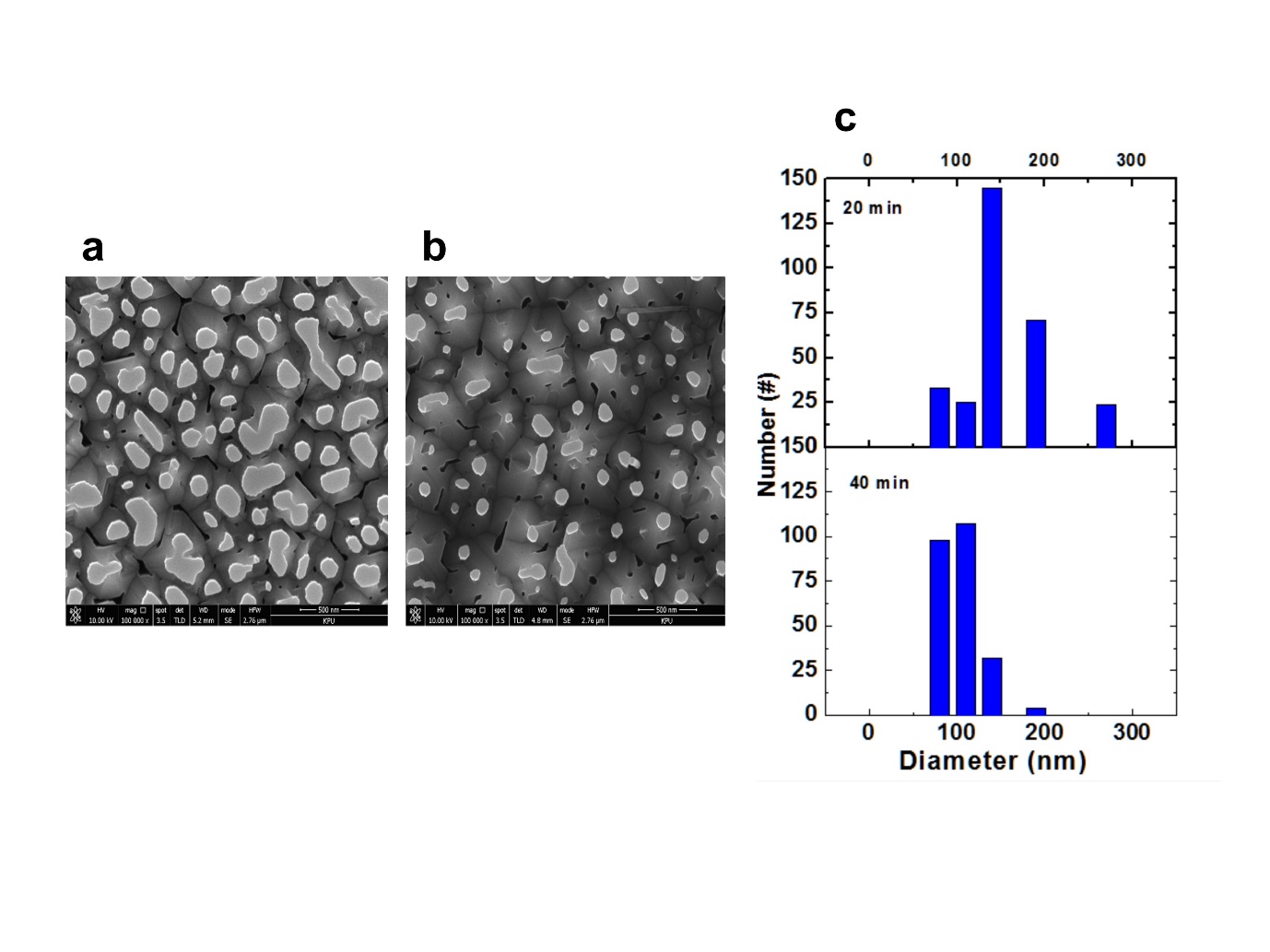


**Figure S1:** The diameter of AlN nanorods calculated from plan-view SEM images decreased from 180 to 95 nm as the etching time was extended from a) 20 min to b) 40 min. c) A slight decrease in the number of AlN nanorods was caused by etching out of AlN nanorods with smaller diameters.


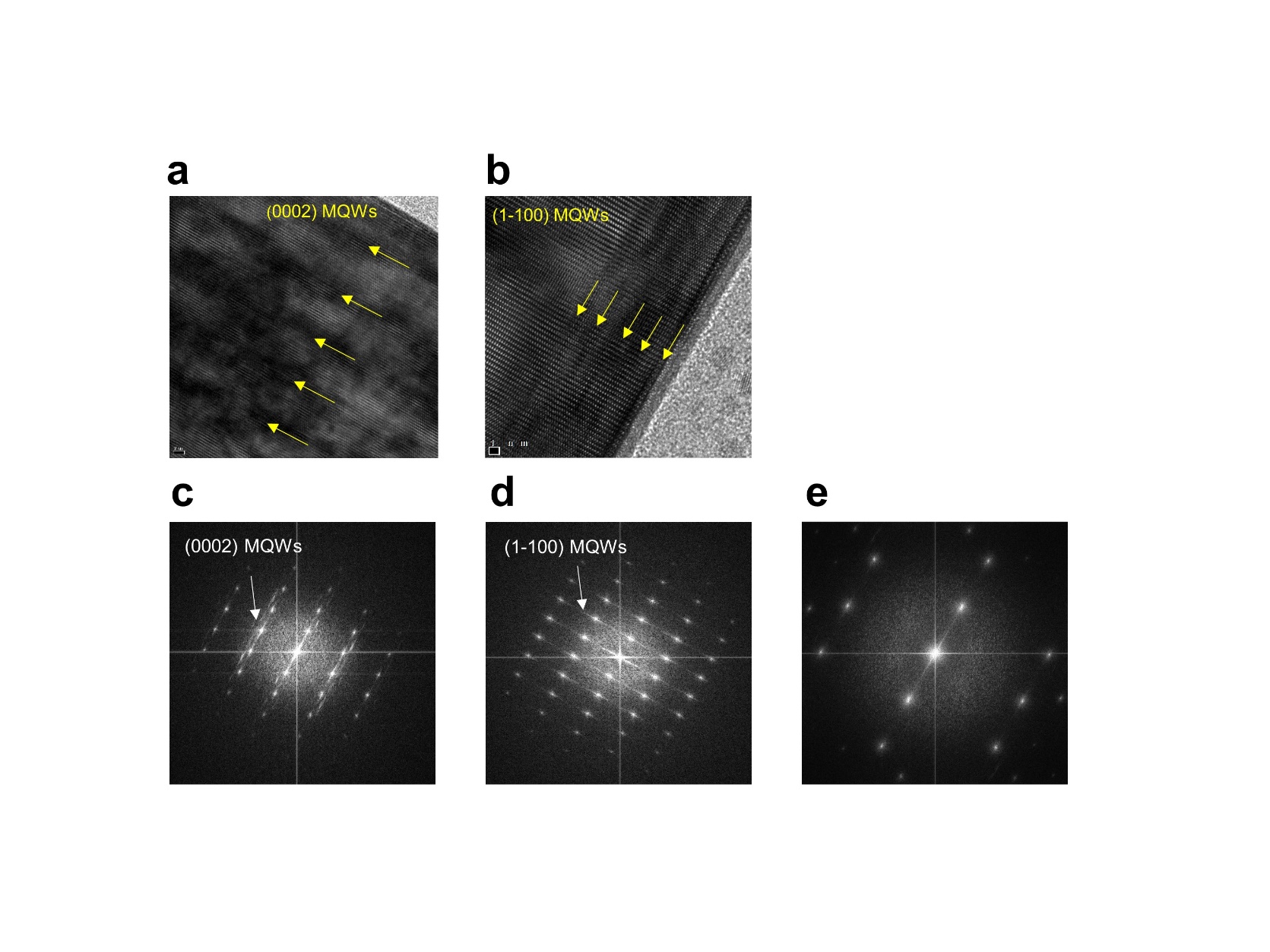


**Figure S2:** HR-TEM images of AlGaN/AlN MQWs on AlN nanorods in the a) [0002] and b) [1-100] directions. Diffraction patterns of c) [0002] and d) [1-100] MQWs taking e) the diffraction pattern of AlN as a reference.


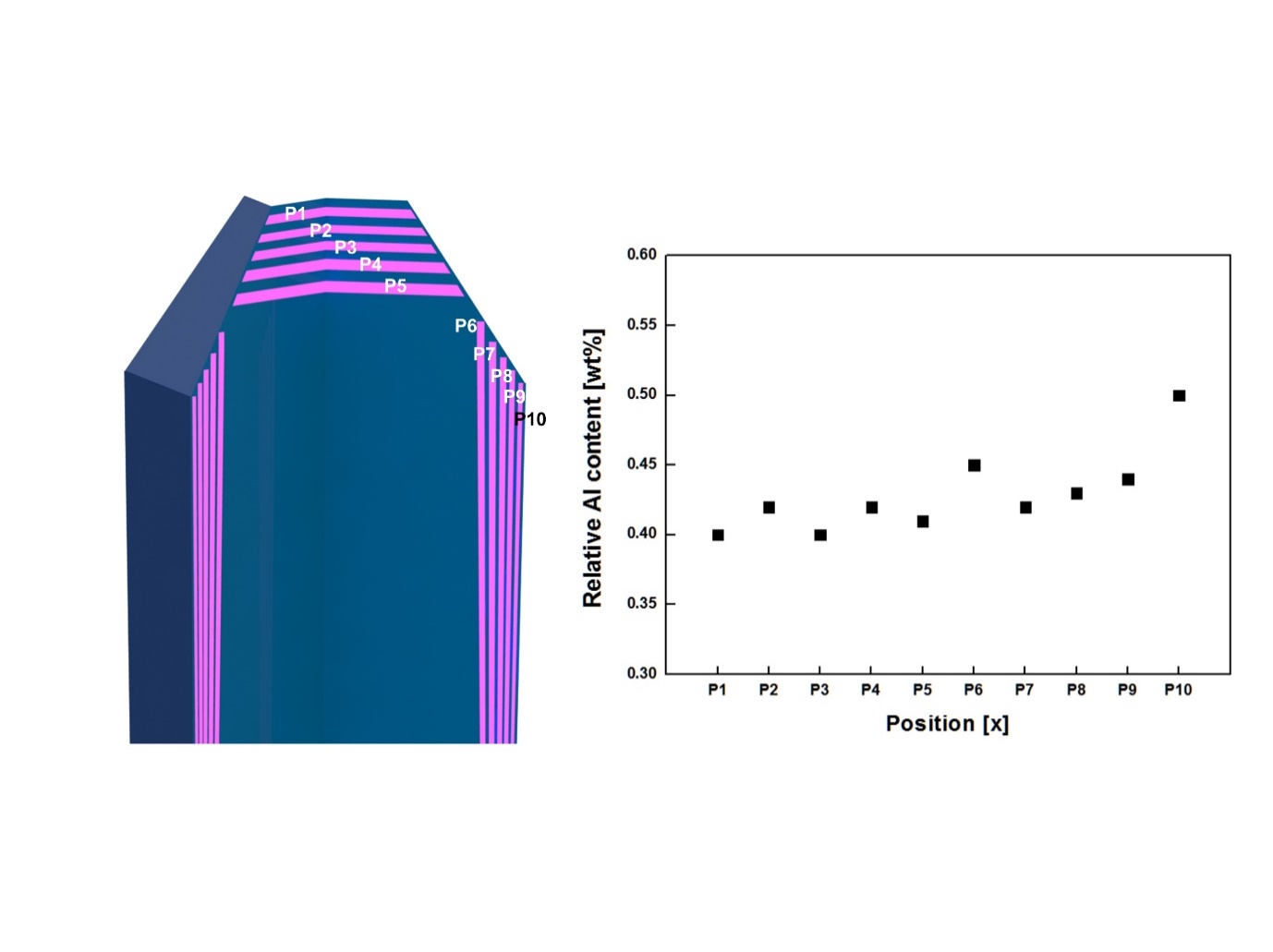


**Figure S3:** Relative Al composition of AlGaN wells along [0002] and [1-100] directions measured by TEM energy-dispersive spectroscopy line scans. The Al composition of the [0002] MQWs is slightly lower owing to the higher migration length of Ga atoms in the c plane. However, the Al contents in the [0002] and [1-100] directions show no significant difference.


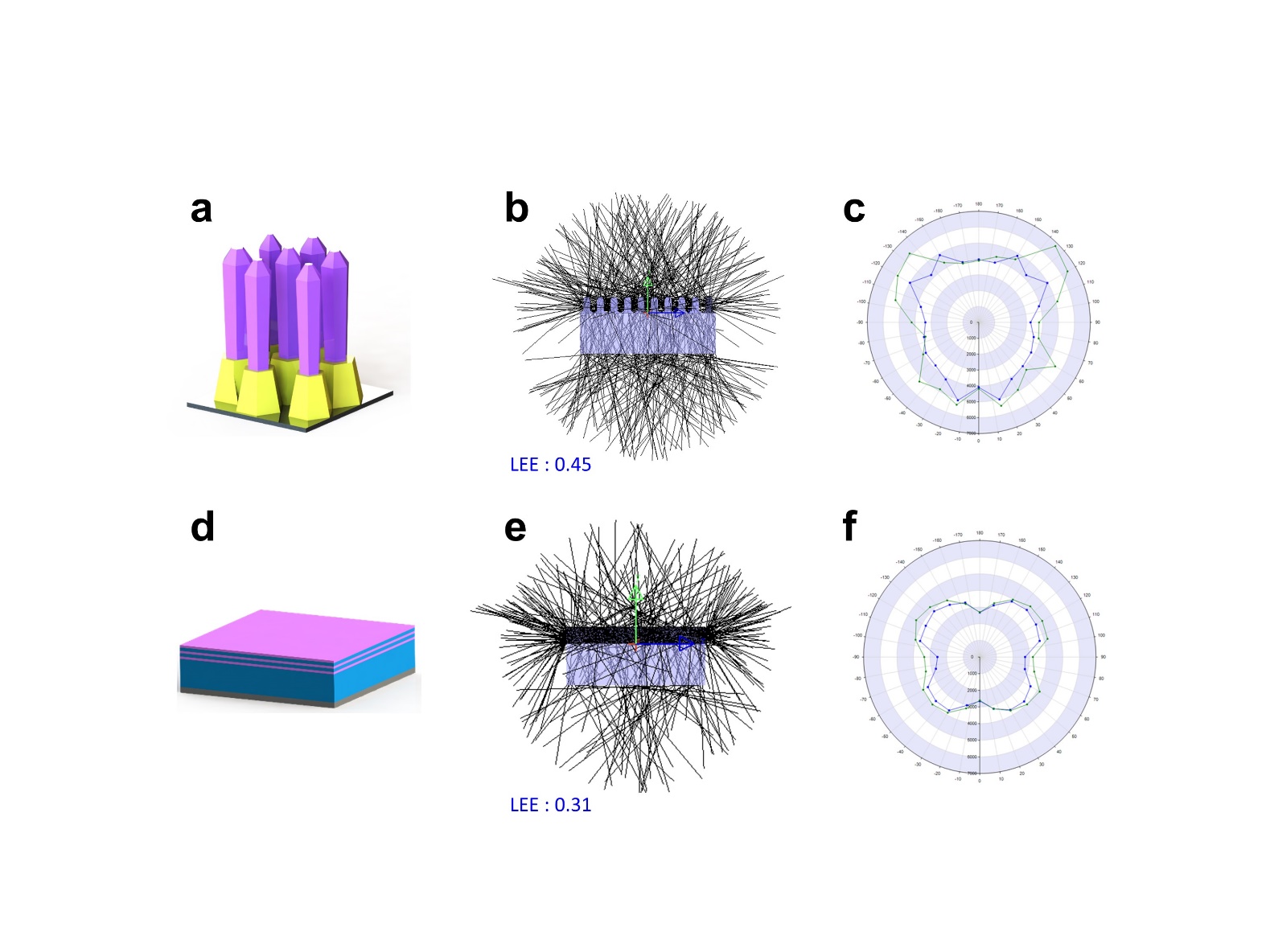


**Figure S4:** Light extraction efficiency of AlGaN/AlN MQWs on AlN nanorods (top row) compared to that of planar MQWs (bottom row), calculated by LightTools simulator. a), d) Schematic images of MQWs on nanorods and conventional planar structure. b), e) Ray tracing of light extraction used to calculate the extraction efficiency. The LEEs of MQWs on the nanorods and conventional planar structure were 0.45 and 0.31, respectively. c), f) Light intensity distributions.
